# Flash Photodynamic Therapy – How the Saturation of Photosensitizer Absorption Enables Selective and Deeper Tumor Treatments

**DOI:** 10.1002/advs.202513199

**Published:** 2025-11-16

**Authors:** Luis G. Arnaut, Fábio A. Schaberle, José Sereno, Lígia C. Gomes‐da‐Silva

**Affiliations:** ^1^ CQC‐IMS Department of Chemistry University of Coimbra Coimbra 3004‐535 Portugal; ^2^ Coimbra Institute for Biomedical Imaging and Translational Research (CIBIT) University of Coimbra Coimbra 3000‐548 Portugal

**Keywords:** photodynamic therapy, pulsed lasers, saturation of absorption, tumor selectivity

## Abstract

FLASH therapies are attracting tremendous interest because they spare normal tissues while maintaining tumor‐destroying efficacy, when compared with continuous delivery of radiation. In Photodynamic Therapy (PDT), it has been noted that continuous‐wave and pulsed lasers give comparable results for a variety of tumors, but the conditions for increased tumor‐to‐peritumoral tissue selectivity have never been reported. This work presents a model that explains how pulsed lasers, in combination with photosensitizers, can offer selective and in‐depth tumor ablation. It is shown that the photosensitizer absorption must be saturated to obtain the FLASH effect. The importance of the number of laser pulses to destroy tumor tissue and spare normal tissue is demonstrated. The predictions of the model and the superiority of FLASH‐PDT are validated with the treatment of subcutaneous CT26 and orthotopic 4T1 tumors models. Notably, FLASH‐PDT with redaporfin significantly increases the overall survival of mice with 4 mm orthotopic 4T1 tumors and lung metastasis. FLASH‐PDT allows for the use of order‐of‐magnitude higher drug or light doses without affecting healthy tissues, while promoting selective and deeper tumor treatments.

## Graphical abstract

FLASH‐PDT uses strongly‐absorbing photosensitizers and pulsed lasers to saturate the photosensitizer absorption at ≈1‐cm tissue depths. This allows for the generation of [ROS] above the necrosis threshold level for tumors, while maintaining [ROS] in peritumoral tissues below that threshold, even when the tumor‐to‐peritumoral tissue ratio is as low as 2. The result is selective and in‐depth tumor treatment.

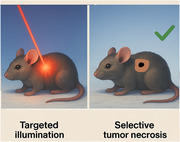

## 1 Introduction

Continuous sources used in radiotherapy (electrons, protons, ionizing photons) or in photodynamic therapy (non‐ionizing photons) are associated with narrow separations between the tumor control probability and the normal tissue complication probability curves. In radiotherapy (RT), the radiation dose that kills normal tissues in only 1.5 to 3 times higher than that required to kill tumor cells.^[^ ^1^ ^]^ The delivery of ultrahigh dose‐rate radiation, named FLASH‐RT,^[^ ^2^ ^]^ was shown to damage tumor cells with the same efficiency as the conventional delivery of radiation, but to spare more normal tissue, allowing for the use of 20–30% higher doses with the same toxicity.^[^ ^3^ ^]^ Differences in reactive oxygen species (ROS) seem to account for differences in FLASH and conventional radiotherapies, but this is still a matter of debate.^[^ ^4^ ^]^ ROS also mediates the photodynamic effect. Continuous and pulsed laser sources have been investigated in PDT, but FLASH selectivity in PDT has never been reported.

PDT uses red/infrared light to excite a photosensitizer molecule in the presence of molecular oxygen (^3^O_2_), to generate ROS and trigger cell death. Photosensitizer development was driven by the maximization of intense electronic transitions in the phototherapeutic window (650–850 nm), where photons with sufficient energy to generate ROS penetrate more deeply in human tissues.^[^ ^5^ ^]^ Singlet states have lifetimes (*τ*_S_) that are too short to react with ^3^O_2_, and ROS precursors are the photosensitizers triplet states.

Solid tumor responses to PDT with continuous‐wave (CW‐PDT) or pulsed lasers were typically compared using the same radiant exposure *H* (units of J cm^−2^, commonly named “light dose”). Animal studies with photosensitizers such as hematoporphyrin derivative (HpD),^[^ ^6^ ^]^ Photofrin (a commercial preparation of HpD),^[^ ^7^, ^8^ ^]^ aluminum sulphonated phthalocyanine (AlSPc),^[^ ^9^ ^]^ or *m*‐tetrahydroxyphenylchlorin (Foscan),^[^ ^10^ ^]^ illuminated by nanosecond lasers with high (> 1 kHz) repetition rates and low fluences per pulse (< 0.2 mJ cm^−2^ per pulse) led to the consensus that these lasers elicit equivalent biological effects as CW lasers. This is readily understood considering that low laser fluences cannot saturate the absorption of photosensitizers. However, nanosecond lasers with low (< 30 Hz) repetition rates and high fluences per pulse (>1 mJ cm^−2^) gave contrasting results. They yielded poor results when AlSPc was employed,^[^ ^9^ ^]^ but gave deeper tissue necrosis^[^ ^11^ ^]^ and better tumor response with HpD.^[^ ^12^ ^]^ Transient saturation of photosensitizer absorption can explain a marginal increase in depth,^[^ ^13^ ^]^ but the depth of necrosis obtained with CW and pulsed lasers was indistinguishable for benzoporphyrin derivative monoacid A (BPD‐MA).^[^ ^14^ ^]^

Skin exposure irradiation limits of CW and pulsed lasers cannot be directly compared because they are expressed in different units.^[^ ^15^ ^]^ At 750 nm, this limit is the irradiance exposure limit *I*_max_≤252 mW cm^−2^ for CW‐lasers, but it is the radiant exposure *E*_L_≤25.2 mJ cm^−2^ per pulse for nanosecond pulsed lasers. Thermal effects occur above these limits. These two limits can be compared introducing the pulse repetition rate *p*_r_. A 25.2‐mJ cm^−2^ pulsed laser reaches the average irradiance of 252 mW cm^−2^ at *p*_r_ = 10 Hz, but its peak irradiance is extremely high: 2.5 MW cm^−2^ (10^17^ photons cm^−2^ at 750 nm) for a 10‐ns laser pulse. Such high photon fluxes may saturate the absorption of chromophore molecules present at micromolar concentrations in tissues, especially those closer to the surface of light incidence. Photons in excess of the saturation limit do not contribute to phototoxicity in the region of saturation. Of note, when the number (*n*_p_) of nanosecond laser pulses exceeds 600, *E*_L_ must be lowered by the correction factor 5×*n*_p_^−0.25^ to avoid thermal effects.

Two major barriers restrict widespread use of PDT: i) attenuation of light in tissues, which limits the depth of the treatment; ii) insufficient photosensitizer selectivity to target tissues, which limits the dose that can be delivered without adverse effects. Here we show that properly designed pulsed lasers dramatically enhance the selectivity of PDT and allow for the use of order‐of‐magnitude higher doses than CW lasers. Pulsed‐laser selectivity and higher light doses can double the depth of the photodynamic effect in target tissues and spare healthy tissue. We name FLASH‐PDT the use of pulsed lasers to saturate the absorption of photosensitizer in PDT.

## 2 Results

### 2.1 Saturation of Absorption

**Figure 1:**
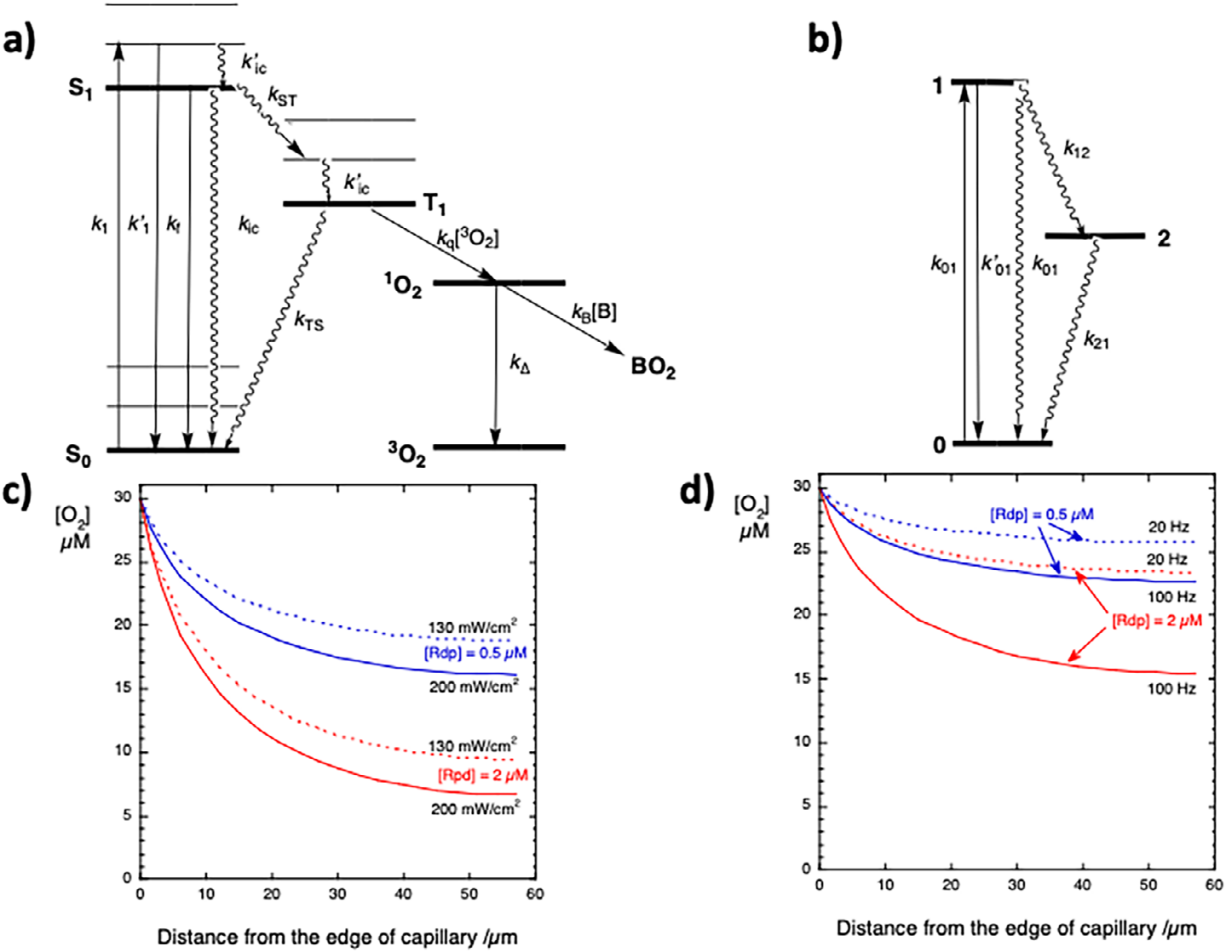
Mechanisms of interaction with molecular oxygen in conventional and FLASH PDT. a) Electronic states involved in the excitation of a photosensitizer and energy transfer to molecular oxygen, and reaction of singlet oxygen (^1^O_2_) with a biomolecule B. b) 3‐State model with the key features required to model the saturation of the absorption of a photosensitizer, where *k*’_01_ is the radiative (fluorescence) rate constant and rate constants are the reciprocal of lifetimes, *k*_i_ = 1/*τ*_i_. c) Steady‐state molecular oxygen concentrations as a function of the distance from the edge of the closest capillary for CW‐PDT, with *H* = 50 J cm^−2^, local irradiances *I*_loc_ = 130 or 200 mW cm^−2^, and local redaporfin concentrations of 2 and 0.5 µm. d) Same as previous caption but for FLASH‐PDT, with *H* = 50 J cm^−2^ and 10 mJ cm^−2^ laser pulses with repetition rates *p*_r_ = 20 or 100 Hz.

Saturation of light absorption occurs when the number of photons absorbed per unit of time in the transition from the ground to the excited state equals the number of excited states decaying per unit of time. In a two‐level system, this is the equality between the rates of absorption and stimulated emission when the populations of the two states become equal. However, PDT photosensitizers have short S_1_ state lifetimes (*τ*_s_ = 1–15 ns), large triplet quantum yields (Φ_T_>0.5) and long triplet lifetimes (*τ*_T_ = 1–10 µs in biological tissues). The metastable triplet state is a sink that allows for the temporary accumulation of excited photosensitizer molecules (**Figure** **1**a). Hence, the saturation of photosensitizer absorption under CW illumination is described by a three‐level system, Figure 1b. This model has a simple solution for the irradiance that saturates absorption under steady‐state conditions^[^ ^16^, ^17^ ^]^

(1) $$I_{sat}^{CW}=\frac{E_{h\nu }}{\sigma_{a}\tau_{S}}\frac{1}{1+Q}$$

Photon energy has the usual form, *E*_hν _= *hc*/λ, and the ground‐state absorption cross‐section is related to the molar absorption coefficient, σ_a_ = 2303ε_max_/*N*_A_, where *N*_A_ is Avogadro's number. The numerical value applies when *ε*_max_ is expressed in M^−1^ cm^−1^ and *σ*_a_ in cm^2^. The consequences of the sink effect imparted by the metastable triplet state are described by the factor

(2) $$Q=\frac{k_{isc}}{k_{f}+k_{ic}+k_{isc}}\frac{\tau_{T}}{\tau_{S}}=\Phi_{T}\frac{\tau_{T}}{\tau_{S}}$$

Considering that photosensitizer triplet state lifetimes are 3 orders of magnitude higher than the corresponding singlet lifetimes, the *Q* factor makes the irradiance required for saturation in a 3‐level system much smaller than that of a 2‐level system. Using the data for porphyrins (HpD, Photofrin), chlorins (BDP‐MA), phthalocyanines (IRDye700DX), and bacteriochlorins (redaporfin), presented in **Table** **1**, with the reasonable estimate of τ_T_ = 5 µs in cells, we obtain saturation irradiances, *I*_sat_>100 W cm^−2^, much higher than the maximum tolerated irradiances (*I*_max_). *Saturation of photosensitizer absorbance will not occur below the thermal threshold when CW lasers are employed*.

**Table 1:** Properties of representative photosensitizers and irradiance (*I*_max_) or radiant exposure limits (*L*_max_), and pulse radiant exposures that saturate their absorption (*L*_sat_).

Name	λ_max_	ε_max_	Φ_F_	τ_S_	Φ_T_	Φ_pd_	*I*_max_	*L*_max_	*L*_sat_
	nm	M^−1^ cm^−1^		ns			mW cm^2^	mJ cm^−2^	mJ cm^−2^
HpD^a)^	620	1500	0.03	15.6	0.63	5 × 10^−5^	200	20	56
BPD‐MA^b)^	686	34 000	0.105	5.2	0.79	6 × 10^−5^	200	20	2.2
IRDye700DX^c)^, ^d)^	686	210 000	0.19	4.4	> 0.55	1 × 10^−6^	200	20	0.36
Redaporfin^e)^	748	125 000	0.138	3.0	0.65	1 × 10^−5^	252	25	0.56

^a)^ Absorption and molar absorption coefficient in cell suspensions,^[^ ^18^ ^]^ fluorescence quantum yield, fluorescence lifetime at pH 7.4,^[^ ^19^ ^]^ Φ_pd_ in buffer of pH 7,^[^ ^20^ ^]^ Φ_∆_ = 0.76 in methanol^[^ ^21^ ^]^;

^b)^ In methanol, Φ_∆_ = 0.76^[^ ^22^ ^]^;

^c)^ ^[^ ^22^ ^]^;

^d)^ In ethanol, Φ_∆_ = 0.55^[^ ^23^ ^]^;

^e)^ Absorption and molar absorption coefficient in CrEL:EtOH:NaCl 0.9%,^[^ ^24^ ^]^ otherwise data in ethanol, Φ_∆_ = 0.43.^[^ ^25^ ^]^

The saturation of the absorption with a laser pulse is readily formulated for a 2‐level system

(3) $$I_{sat}^{PL}=\frac{E_{h\nu }}{\sigma_{a}\tau_{1}}$$

where *τ*_1_ is the lifetime of the upper level. If the pulse duration is much longer than this lifetime, the saturation behavior is essentially the same as for CW lasers. However, if the laser pulse duration *τ*_p_ is much shorter than *τ*_1_, then the saturation radiant exposure is^[^ ^26^ ^]^

(4) $$L_{2-sat}=\frac{E_{h\nu }}{2\sigma_{a}}$$

The radiant exposure, rather than the irradiance, determines the saturation behavior. The change in population of one level causes an equal and opposite change in population in the other level, and requires the division by 2.

The population of a 3‐level system (Figure 1b) where *τ*_p_ is much shorter than *τ*_1_ but much longer than *τ*_2_, is driven to level 2 and remains there during the pulse. The saturation radiant exposure is now given by^[^ ^26^ ^]^

(5) $$L_{3-sat}=\frac{E_{h\nu }}{\sigma_{0}+\sigma_{1}}$$

In the case of a photosensitizer, σ_1_ refers to the triplet state and can be neglected at the excitation wavelength in comparison with σ_a_, which is σ_0_ in the expression above. Consequently, a reasonable approximation for the saturation fluence in FLASH‐PDT is^[^ ^27^ ^]^

(6) $$L_{sat}=\frac{E_{h\nu }}{\sigma_{a}}$$

Table 1 presents the limit of saturation radiant exposure for various photosensitizers under nanosecond laser pulse excitation, together with the maximum fluence exposure at the corresponding *λ*_max_.

Under saturation, the local ROS concentration generated with one laser pulse cannot exceed the local concentration of the photosensitizer. The actual amount to ROS generated depends on the local concentration of oxygen and the number of laser pulses.

### 2.2 Concentration of Molecular Oxygen in Tissues

PDT causes tissue necrosis when the local combination of light, photosensitizer, and oxygen generates a concentration of ROS, most often singlet oxygen (^1^O_2_), above a threshold value, [ROS]_th_. The phototherapeutic threshold dose required to produce tissue necrosis is similar for different photosensitizers, *T*_th_≈10^19^ photons cm^−3^,^[^ ^28^, ^29^ ^]^ and corresponds to a local cumulative amount of activated photosensitizer *C*_loc_^*^≈17 mm. [ROS]_th_ is related to *C*_loc_^*^ through the efficiency of ROS generation: Φ_T_ *τ*_T_ *k*_q_[O_2_]. Using typical values (Tables 1 and **2**), we obtain [ROS]_th_ = 3500×[O_2_]*C*_loc_^*^, where the conversion factor is in units of M^−1^. We need the local [O_2_] to obtain [ROS]_th_.

**Table 2:** Common properties of photosensitizers and tissues.

Definition	Symbol	value	Units
Triplet lifetime in tissues	τ_T_	5	µs
Triplet quenching rate constant by O_2_ in tissues	*k*_q_	10^9^	M^−1^ s^−1^
Oxygen diffusion in tissues	*D*	2 × 10^−5^	cm^2^ s^−1^
First‐order metabolic consumption rate of oxygen	*k*_met_	0.057	s^−1^
Oxygen concentration outside a capillary in normal tissues	[O_2_]_0_	30	µM
Radius of capillary	*r*_0_	3	µm
Intercapillary distance	2*r*_1_	120	nm
Reduced scattering coefficient of tissues at 500 nm	*µ*_s_’(500 nm)	18.9	cm^−1^
Scattering power	*B*	1.286	
Absorption coefficient of tissues	*µ*_a_	0.6	cm^−1^

The concentration of oxygen in tissues is heterogenous and dynamic. The lower limit of physoxia is [O_2_]_0_ = 30 µm.^[^ ^30^ ^]^ We take this as the initial concentration of O_2_ just outside the capillaries. While O_2_ is consumed by metabolism and photodynamic action, new O_2_ molecules diffuse to the tissues. The relevant diffusion to keep tissues oxygenated during PDT is that of O_2_ from capillaries to adjacent issues. Krogh modeled this diffusion, describing each capillary by a cylinder with constant [O_2_] and the surrounding tissue by a concentric cylinder where oxygen consumption does not depend on local [O_2_] (zero‐order reaction).^[^ ^31^ ^]^ The analytical solution for the steady‐state concentration of oxygen [O_2_]_ss_ in the surrounding tissue was used to estimate the zone of oxygen depletion around an isolated capillary during PDT.^[^ ^32^ ^]^ Kirkpatrick presented a steady‐state solution for the case of oxygen diffusion with first‐order oxygen consumption (rate constant *k*_1_) and the same boundary conditions as Krogh's cylinder.^[^ ^33^ ^]^ Kirkpatrick's solution, adopted here, is presented in the Supporting Information and implemented in a spreadsheet accessible as Supporting Information. Each capillary is regarded as a cylinder with radius *r*_0_ = 3 µm, and the intercapillary distance is taken as 2*r*_1_ = 120 µm.

Figure 1c,d shows [O_2_]_ss_ as a function of the distance *r* from the capillary for various irradiances and redaporfin concentrations. [Rdp]_loc_ = 2 µm is representative of tumors, and [Rdp]_loc_ = 0.5 µm is representative of peritumoral tissues, and all tissues are considered well‐oxygenated tissues ([O_2_]_0_ = 30 µm). When [Rdp] = 2 µm and the oxygen‐consumption rate constant increases to *k*_1_≈1.3 s^−1^ (*I*_loc_ = 200 mW cm^−2^), [O_2_]_ss_ drops to 10 µm at *r* = 25 µm in the tumor, and hypoxia is probably unavoidable. Note that these calculations do not account for the contribution of another capillary 114 µm from the edge of the capillary considered. Oxygen depletion in tumors may limit CW‐PDT at irradiances below *I*_max_. However, FLASH‐PDT is not normally affected by oxygen depletion for *p*_r_≤100 Hz, and it does not depend on the radiant exposure of the laser pulse (*L*_0_). Only exceedingly high pulse repetition rates make [O_2_]_ss_ drop below 10 µm. The choice of pulsed lasers with *p*_r_ values below 100 Hz is mostly a matter of convenience. Very low repetition rates (e.g., *p*_r_ = 1 Hz) require long treatment times, whereas lasers with high *p*_r_ are more expensive.

Assuming that *T*_th_ was determined in conditions were [O_2_]_ss_>10 µm, we obtain [ROS]_th_>0.6 mm as the limit to induce tissue necrosis. [ROS]_th_ between 0.5 and 1.0 mM have been reported for Photofrin, Foscan, BPD,^[^ ^34^, ^35^, ^36^ ^]^ and redaporfin,^[^ ^29^ ^]^ at different drug‐to‐light intervals (DLI). In this work, we consider that tissue necrosis occurs if [ROS]≥0.7 mm in all the regions between capillaries at a given depth *d* from the illuminated surface.

### 2.3 Concentration of ROS in Tissues

PDT generates ROS from oxygen, and the rate of ROS generation is related to the rate of oxygen consumption. Considering the attenuation of light in tissues and photobleaching, we obtain (see Supporting Information)

(7) $${\left[\mathrm{ROS}\right]}_{c}=1240\frac{\lambda \varepsilon }{hcN_{A}}\Phi_{T}\tau_{T}k_{q}{\left[O_{2}\right]}_{ss}\frac{C_{loc}^{0}}{k_{pd}}\left(1-e^{-k_{pd}t}\right)b_{s}I_{0}e^{-d/\delta }$$

for CW‐PDT, where [O_2_]_ss_ is the steady‐state concentration of oxygen at the distance from the closets capillary at which [ROS] is calculated, *C* ^0^_loc_ is the initial local concentration of the photosensitizer, *k*_pd_ its photodecomposition rate constant, *t* the duration of illumination with irradiance *I*_0_ at the surface of the tissue, *d* the depth from the surface at which [ROS] is evaluated, and the other parameters are presented in Table 2. This equation is implemented in spreadsheets presented in the Supporting Information. Its evaluation requires parameters of the photosensitizers (Table 1) and the tissues (Table 2), the local [O_2_], and the illumination conditions (*I*_0_, *t*, *H* = *I*_0_ *t*).

The rate of photon absorption in FLASH‐PDT is limited by *C*_loc_ because all photosensitizer molecules are excited when absorption saturates. Under similar conditions as above, we obtain (see Supporting Information)

(8) $${\left[\mathrm{ROS}\right]}_{f}=\Phi_{T}\tau_{T}k_{q}{\left[O_{2}\right]}_{ss}C_{loc}^{0}{\left(1-\Phi_{pd}\right)}^{n_{p}/2}n_{p}$$

for FLASH‐PDT, where *n*_p_ (= *H*/*L*_0_) is the number of laser pulses. This equation applies only to the regions where photosensitizer absorption is saturated, and in these regions [ROS]_f_ does not dependent on light intensity. *L*_loc_ will drop below *L*_sat_ at a sufficiently large depth of light penetration, and the calculation of [ROS] shifts to the equation for CW illumination, except that *I*_0_ is replaced by *L*_0_ *p*_r_.

*C* ^0^_loc_ is in the µm range for tumors and highly‐vascularized tissues.^[^ ^29^, ^34^, ^37^, ^38^ ^]^ However, *C* ^0^_loc_ in tumors with elevated interstitial fluid pressures may drop to the nm range.^[^ ^39^ ^]^ Photosensitizer tumor‐to‐peritumoral ratios depend on DLI and drug formulation, in addition to photosensitizer and tumor properties. Typical ratios for HPD analogues and for BDA‐MA are ≈5 and ≈2, respectively.^[^ ^40^ ^]^ Redaporfin offers tumor‐to‐muscle ratios of ≈2 or ≈4 for DLI = 15 min or 24 h, respectively.^[^ ^41^ ^]^

**Figure 2:**
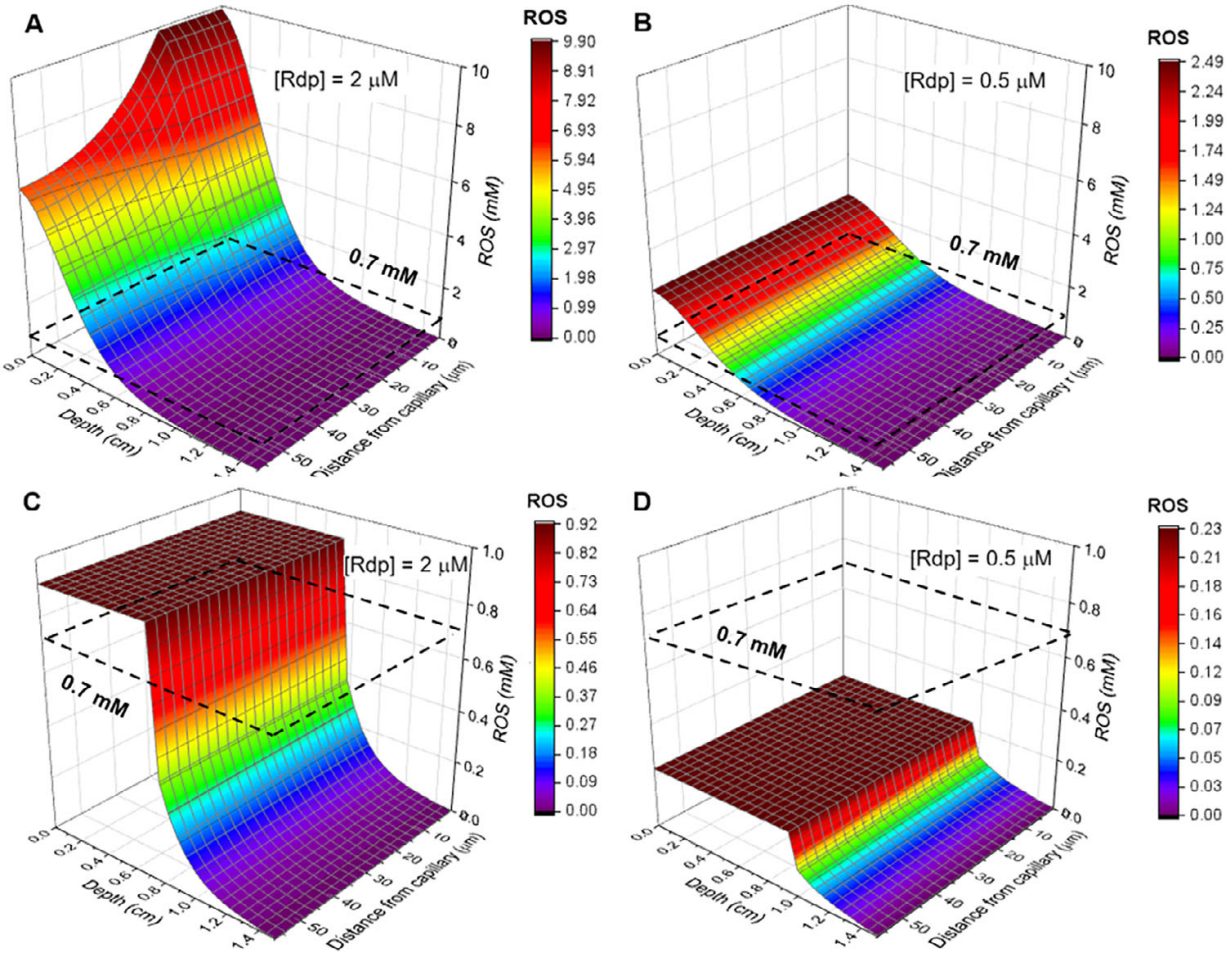
Dependence of the concentration of reactive oxygen species on the depth from the surface of the tissue and on the distance from the closest capillary. a) CW‐PDT with *I*_0_ = 50 J cm^−2^ at 130 mW cm^−2^ and local concentration of redaporfin [Rdp]_loc_ = 2 µm. b) CW‐PDT under the same conditions but [Rdp]_loc_ = 0.5 µm. c) FLASH‐PDT with *I*_0_ = 50 J cm^−2^ and *L*_0_ = 10 mJ cm^−2^ at 20 Hz, and [Rdp]_loc_ = 2 µm. d) FLASH‐PDT under the same conditions but [Rdp]_loc_ = 0.5 µm.

**Figure** **2**a,b shows that [ROS]_c_ decreases exponentially with the depth of light penetration and follows the decrease of [O_2_] as the distance from the capillary increases. The threshold for necrosis ([ROS]_th_ = 0.7 mm) is also illustrated. A tumor necrosis depth *d*_n_ = 6 mm, calculated with *I*_0_ = 130 mW cm^−2^, *H* = 50 J cm^−2^, and *C*_loc_ = 2 µm, will be associated with a peritumor necrosis depth *d*_n_ = 5 mm if the tumor‐to‐peritumor ratio is 4. The value of *d*_n_ is determined by the condition [ROS]_th_>0.7 mm at *r*_1_ when [O_2_]_ss_ is as twice the value Kirkpatrick's solution at this point, capped to the maximum [O_2_]_ss_ = 30 µm. The poor selectivity of CW‐PDT is, in part, due to the good oxygenation of normal tissues maintained at *C*_loc_ = 0.5 µm.

Figure 2c,d reveals that [ROS]_f_ is independent of depth as long as the absorption of the photosensitizer is saturated, and independent of the distance to capillaries because good oxygenation ([O_2_]_ss_ = 30 µm) is always maintained. This leads to extended plateaus of [ROS]_f_ whose heights depend of *C*_loc_ and *n*_p_. Using *L*_0_ = 10 mJ cm^−2^ at 20 Hz, *H* = 50 J cm^−2^, and *C*_loc_ = 2 µm, we have a necrosis depth of *d*_n_ = 6.5 mm. The calculated tumor necrosis depths of CW‐PDT and FLASH‐PDT are indistinguishable, which explains the equivalence between CW and pulsed‐laser PDT reported in earlier studies. However, it has not been remarked that tissues with lower *C*_loc_ are spared in FLASH‐PDT.

The sudden drop in [ROS] illustrated in Figure 2c (and in Figure 2d) corresponds to the depth at which *L* becomes lower than *L*_sat_ (Table 1). The value of *L* depends on the intrinsic attenuation of light in tissues and on the absorption of light by the photosensitizer. This explains why the drop occurs at greater depth in Figure 2d: the concentration (absorption) of the photosensitizer is lower.

Photosensitizer distribution in tumors is heterogeneous.^[^ ^42^ ^]^ Photosensitizer molecules may be confined in the vasculature for short DLI, parallel the distribution of oxygen for longer DLI, or accumulate in the periphery of tumors when they have high solid stress.^[^ ^39^ ^]^ This introduces uncertainty in *C* ^0^_loc_ and, consequently in [ROS] calculations. This is a general problem and may compromise therapeutic efficacy. Nevertheless, **Table** **3** collects representative depths of necrosis reported for HPD, BPD‐MA, and redaporfin and show that they are reasonable well reproduced by our model, without adjustable parameters.

**Table 3:** Observed and calculated tissue necrosis depths for CW‐PDT (*I*_0_) or FLASH‐PDT (*L*_0_).

Name	λ_max_	Tumor	Dose	DLI	*C* ^0^_loc_	*H*	*I*_0_	*L*_0_	[ROS]^a)^	d_n_
	nm		mg kg^−1^	h	µm	J cm^−2^	mW cm^2^	mJ cm^2^	mm	mm
Photofrin^b)^	630	RIF	5	18–24	5.0 ± 1.6	50	150		obs: 0.3	
									calc: 0.24	
Photofrin^b)^	630	RIF	5	18–24	4.4 ± 0.2	250	75		obs: 1.4	
									calc: 0.9	
Photofrin^c)^	630	RIF	10	24	10	30	75			obs: 1.5 ± 0.2
										calc: 1.1
BPD‐MA^d)^	690	R‐3327	2	1	2^e)^	50	200			obs: 8.7 ± 2.5
										calc: 5.0
Redaporfin^f)^	750	liver^g)^	0.75	0.25	2	5	130			obs:1.6
										calc: 1.5
Redaporfin^f)^	750	liver^g)^	0.75	0.25	2	10	130			obs: 2.5
										calc: 2.5
Redaporfin^f)^	750	liver^g)^	0.75	0.25	2	25	130			obs: ≈4
										calc: 4.0
BPD‐MA^d)^	690	R‐3327	2	1	2^e)^	50		12.5		obs: 7.7 ± 2.3
										calc: 6
BPD‐MA^d)^	690	R‐3327	2	1	2^e)^	50		22.5		obs: 8.5 ± 1.8
										calc: 7

^a)^ [ROS] at 3 mm depth;

^b)^ Ref. [36];

^c)^ Ref. [43];

^d)^ Ref. [14];

^e)^ Estimated using *C* ^0^_loc_ = 60 µm for liver after 1 h of 5 mg kg^−1^ administration of BPD‐MA,^[^ ^14^ ^]^ and a liver‐to‐tumor concentration ratio of 4 in mice 3 h after BDP‐MA administration^[^ ^44^ ^]^;

^f)^ Ref. [29];

^g)^ Healthy tissue.

### 2.4 Selectivity and Depth of Necrosis with FLASH‐PDT

**Figure 3:**
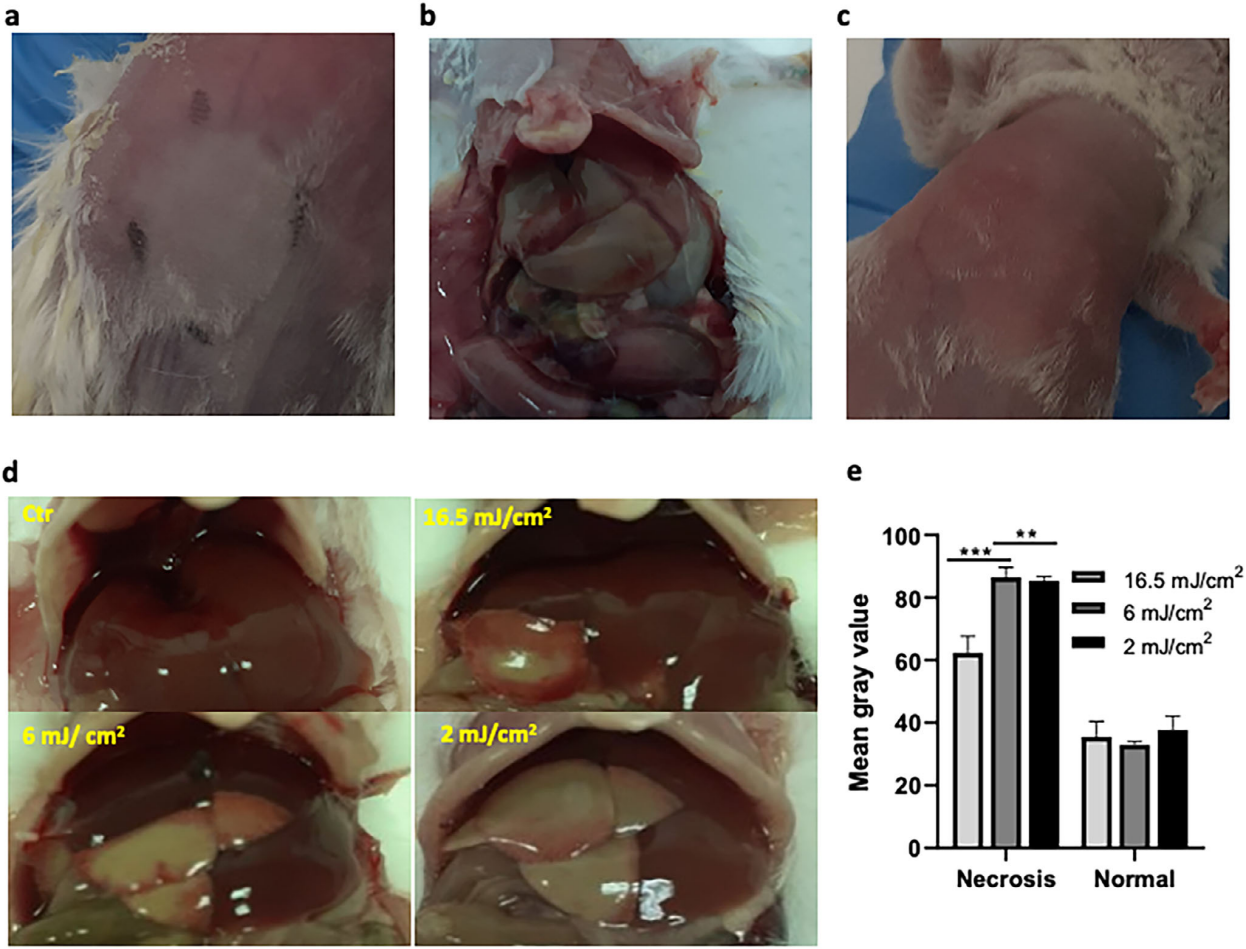
Illumination (*H* = 30 J cm^−2^) of the liver of healthy BALB/c 15 min after administration of 0.9 mg kg^−1^ of redaporfin. a) Image of the area illuminated with CW laser (30 J cm^−2^, 130 mW cm^−2^) showing skin damage at 72 h post‐illumination. b) Image of the liver and surrounding tissues after CW illumination. c) Image of the area illuminated with pulsed laser (30 J cm^−2^ using laser pulses of 5 mJ cm^−2^) showing no skin damaged at 72 h post‐illumination. d) Representative images of the liver of the animals in FLASH‐PDT (30 J cm^−2^ using laser pulses of 16.5, 6, or 2 mJ cm^−2^). e) Image‐J treatments of regions of interest in the necrotic and normal liver showing that the highest pulse fluence leads to the smaller necrosis (*n* = 4).

In FLASH‐PDT with a set light dose (e.g., *H* = 30 J cm^−2^), [ROS]_f_ increases with the number of pulses and eventually changes the biological impact from none to necrosis. This was tested illuminating mice with CW or pulsed lasers, in the region of the liver, through the skin, 15 min after the administration of 0.9 mg kg^−1^ of redaporfin. We estimate *C* ^0^_loc_≈2.4 µm in the liver in these conditions based on *C*_loc_ = 2 µm after administration of 0.75 mg kg^−1^ redaporfin.^[^ ^29^ ^]^ Two of the three mice illuminated with the CW laser (*I*_0_ = 105 mW cm^−2^) died from necrosis in various organs (**Figure** **3**a,b). This was not unexpected because we calculated *d*_n_ = 4.5 mm for liver (*C* ^0^_loc_≈2.4 µm) and 4.0 mm for nearby tissues (*C* ^0^_loc_≈0.6 µM). Figure 3b confirms indiscriminate damage in the liver region after CW‐PDT. However, FLASH‐PDT with the same drug and light doses but using *L*_0_ = 2, 6, or 16 mJ cm^−2^ spared the skin (Figure 3c) but produced liver necrosis (Figure 3d) without significant damage to nearby tissues (Figure 3e). We calculate [ROS]_f_ = 3.1, 1.1, and 0.4 mm for *C* ^0^_loc_≈2.4 µm, as the laser pulse fluence increases from 2 to 6 and 16.5 mJ cm^−2^. Liver necrosis is expected for *L*_0_ = 2 and 6 mJ cm^−2^. Figure 3d,e indeed shows that more laser pulses (lower light pulse fluences) give larger liver necroses. Although the total light dose is the same (*H* = 30 J cm^−2^), more pulses with lower *L*_0_ place larger tissue areas above the necrosis threshold ([ROS]_th_>0.7 mm). Each laser pulse adds a quantity of ROS proportional to *C* ^0^_loc_, and the cumulative ROS only reaches [ROS]_th_ after a sufficiently large number of pulses, provided that the absorption of the photosensitizer is saturated. In the regions where [ROS]_th_ is attained, adding more pulses does not change the result, which explains the similarity between *L*_0_ = 2 and 6 mJ cm^−2^.

The liver/intestine ratio of redaporfin is 6 at DLI = 15 min.^[^ ^41^ ^]^ With *C* ^0^_loc_≈0.4 µm and the laser pulse fluence 2, 6, and 16.5 mJ cm^−2^, we calculate [ROS]_f_ = 0.5, 0.2, and 0.07 mm for the intestine, respectively, and we do not expect damage. The same applies for the lungs and skin (liver/lungs = 10, liver/skin = 6). Kidneys and spleen, both with ratios of 2 at this DLI,^[^ ^41^ ^]^ could be damaged. Figure 3c,d confirms that most tissues next to the liver are indeed spared, which explains the survival of all mice. *These experiments confirm that FLASH‐PDT spares tissues with lower C^0^_loc_, which increases safety and selectivity, and enables the use of higher doses*.

### 2.5 FLASH‐PDT of Large CT26 Tumors and of Orthotopic 4T1 Tumors

**Figure 4:**
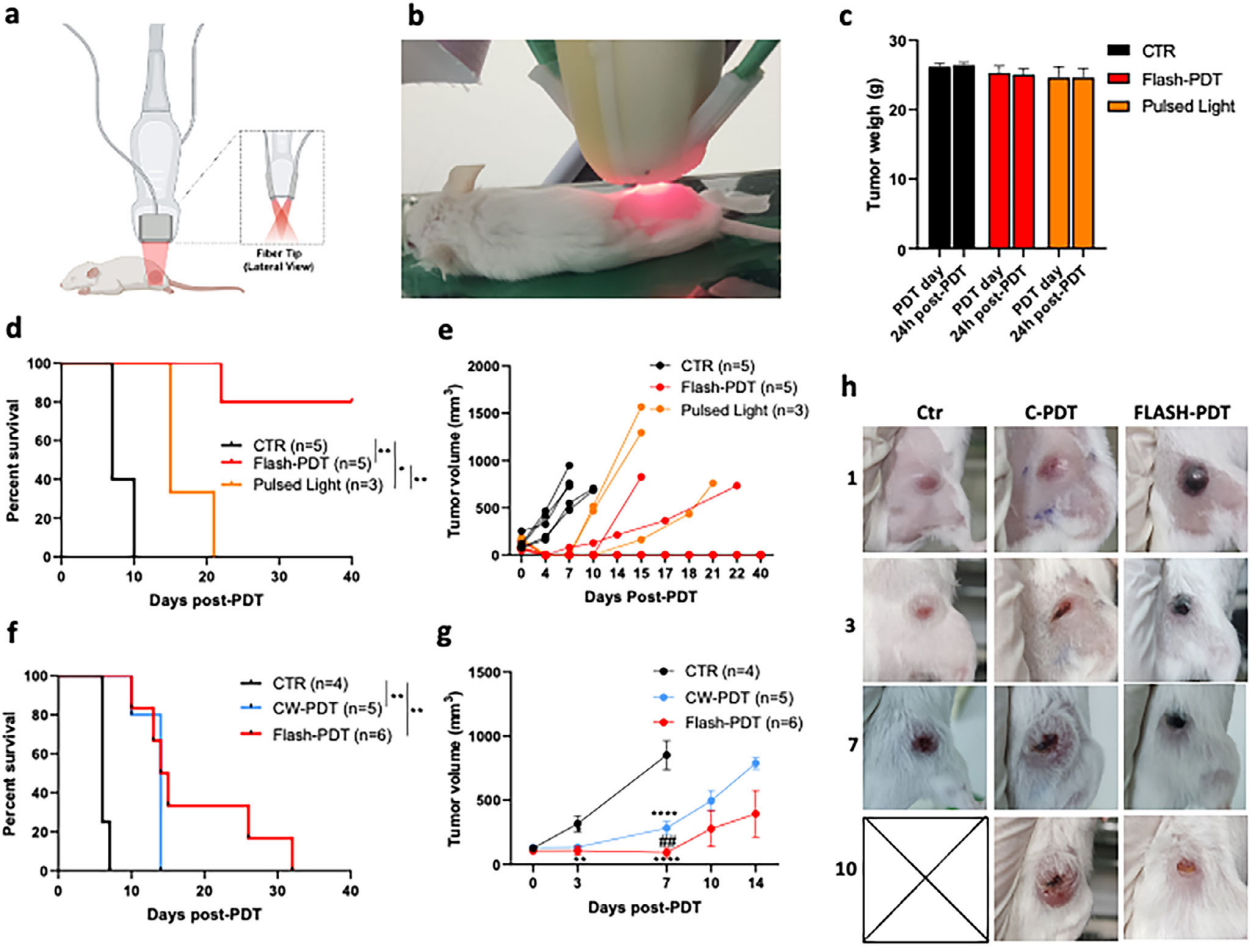
Treatment of subcutaneous CT26 tumors 15 min after administration of 0.75 mg kg^−1^ redaporfin. a) Setup used for 750‐nm pulsed laser illumination of a tumor‐centered 13 mm diameter circle. b) Image of tumor illumination. c) Mice weigh before and 24 h after tumor illumination. d,e) Survival and tumor growth curves (≈5 mm tumors at the time of treatment) treated with FLASH‐PDT (432 J cm^−2^) or control conditions, including a pulsed light‐only control (432 J cm^−2^). f,g) Survival and tumor growth curves (≈7 mm tumors at the time of treatment) with either FLASH‐PDT (432 J cm^−2^) or CW‐PDT (50 J cm^−2^). h) Representative images of ≈7 mm tumors at 1, 3, 7, and 10 days post‐treatment with CW‐PDT (50 J cm^−2^, 130 mW cm^−2^) or FLASH‐PDT (432 J cm^−2^, 24 mJ cm^−2^ laser pulses at 20 Hz), Ctr = untreated mice.

Large tumors are notoriously difficult to treat, both because of light attenuation and of the larger amount of healthy tissue in the margins of the treatment. **Figure** **4**a,b illustrates treatments of ≈5 and ≈7 mm subcutaneous CT26 tumors increasing the light dose from 50 J cm^−2^ in CW‐PDT to 432 J cm^−2^ in FLASH‐PDT, 15 min after the administration of 0.75 mg kg^−1^ redaporfin. The illumination field ≈1.4 cm^2^ required to treat 7 mm tumors is lethal for CW‐PDT with light doses > 50 J cm^−2^.^[^ ^24^ ^]^ Figure 4c–e shows that all the animals survived FLASH‐PDT with 432 J cm^−2,^ and 80% were cured without weight loss. We extended the predicted depth of absorption saturation to *d*_n_ = 8 mm, making *L*_0_ = 24 mJ cm^−2^, which is above the threshold for thermal effects for the number of pulses employed. We now have both thermal and photodynamic effects, but Figure 4d shows that the thermal effects of pulsed light alone are not curative. CW‐PDT, with its maximum tolerated light dose has a modest impact in ≈7 mm tumors, but FLASH‐PDT, with its higher maximum tolerated light dose improves tumor responses (Figure 4f–h).

**Figure 5:**
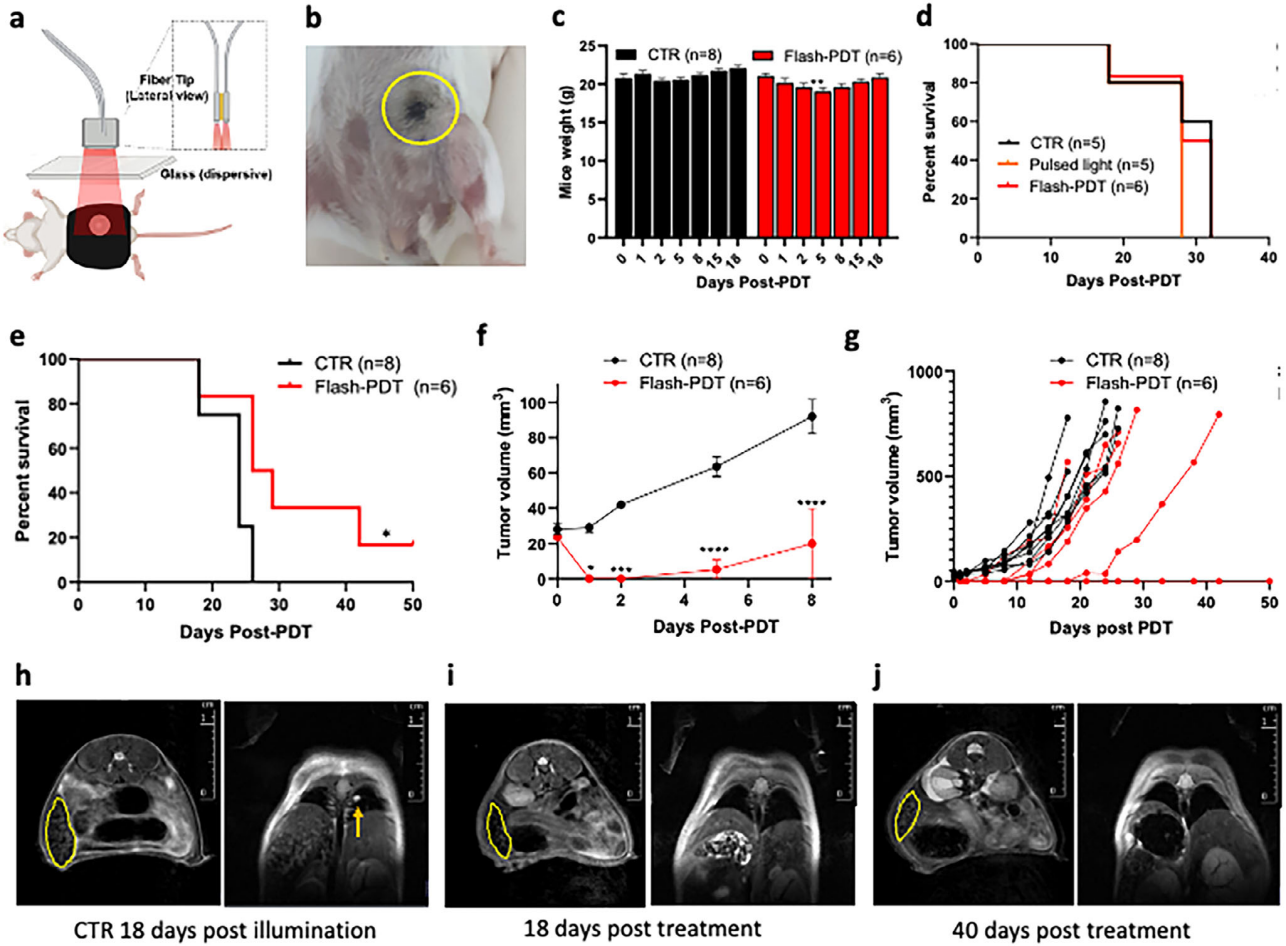
FLASH‐PDT of orthotopic 4T1 tumors with ≈4 mm diameters, 15 min after readporfin administration. a) Illustration of the Vevo LARZ imaging platform optical fibers alignment, a dispersive glass, and a mask demarcating the illumination area. b) Photo of the 12 mm illuminated area 2 days post FLASH‐PDT. c) Weight of control and treated mice. d) Survival plots for treated (8 mg kg^−1^, laser pulses of 12 mJ cm^−2^ to deliver 90 J cm^−2^) and control animals, including control with pulsed light only. e–g) Survival plots and tumor growth kinetics for treated (15 mg kg^−1^, laser pulses of 11 mJ cm^−2^ to deliver 160 J cm^−2^) and control animals. h–j) Magnetic resonance imaging of the spleen (delimitated in yellow) and lungs of control and cured animals, taken 18 and 40 days post‐treatment, where the arrow points to lung metastasis.

Orthotopic 4T1 tumors are extremely difficult to cure because of their high solid stress, immunosuppressive microenvironment, early onset of lung metastasis, and location near vital organs. Growth‐induced solid stress is responsible for a low tumor‐to‐peritumoral redaporfin ratio in these tumors, they do not respond to immune‐checkpoint inhibitors, and tumor resection ≈10 days after the inoculation of 20 000 4T1 cells in the mammary pad leads to lung metastasis ≈30 days later.^[^ ^39^ ^]^ Redaporfin CW‐PDT alone or combined with immune‐checkpoint inhibitors was unable to cure mice with orthotopic 4T1 tumors when they reached ≈4 mm.^[^ ^39^ ^]^ The low accumulation of redaporfin in these tumors motivated the increase of photosensitizer and light doses first to 8 mg kg^−1^ and 114 J cm^−2^ (10 mJ cm^−2^ per pulse) and then to 15 mg kg^−1^ and 160 J cm^−2^ (11 mJ cm^−2^ per pulse), which are below the thermal effects limit, with the setup of **Figure** **5**a. Two days after FLASH‐PDT, necrosis is centered in the tumor, and healthy tissues in the margin are spared (Figure 5b). All animals survived these high doses with minor weigh losses (Figure 5c). All animals responded to the highest‐dose treatment (Figure 5d–g), and there is a statistically significant increase of survival. The spleen of the untreated animals is much increased and lung metastases are present (Figure 5h), but the spleen of cured animals is back to normal size 40 days post‐treatment, and lung metastases were not found (Figure 5i,j). Enhancing the overall survival of animals with metastasized 4T1 tumors is exceptional and was achieved with one single FLASH‐PDT session. *FLASH‐PDT increases the selectivity of PDT and allows for the increase of the tolerated drug and light doses, ultimately leading to higher treatment efficacy and safety*.

### 2.6 Limitations

Pulsed lasers generate a plateau of [ROS] in the tissue regions where photosensitizer absorption is saturated. We showed that the height of this plateau depends on the number of laser pulses and on the local concentrations of photosensitizer and oxygen molecules. Human tumors frequently have oxygen concentrations below those of normal tissues. An oxygenation reduction factor can be introduced in the model to account for hypoxia. This obviously limits the success of the treatments, but we showed that oxygen depletion during the treatment can be avoided in FLASH‐PDT using laser pulse repetition rates below 100 Hz.

A limitation in our model is that it does not take into account the time‐dependence of the increase in the optical penetration depth with the bleaching of the photosensitizer during illumination. In order to account for the change in optical penetration depth with illumination, we used the value calculated for half the illumination time. There is often a small mismatch between λ_max_ and the laser employed in PDT. For simplicity, our calculations employed the laser wavelength rather than λ_max_. These approximations of our model do not obscure the fact that it provides realistic estimates of the depth of necrosis on the basis of simple mathematical relations that use properties of photosensitizers and tissues, and no adjustable parameters. It allows for a direct comparison between CW‐ and FLASH‐PDT, and for the planning of treatments using a simple spreadsheet.

A limitation in our experimental setup is the use of the laser source of a Vevo LAZR photoacoustic imaging equipment rather than of a laser designed for FLASH‐PDT. A proper pulsed laser would allow for a better definition of the illumination field, and the results reported here must be regarded as the basic results that can be achieved with FLASH‐PDT. The use of this laser source does have the virtue of illustrating the simplicity of the transition between CW and pulsed lasers in PDT, because photoacoustic imaging with comparable lasers was already approved by the FDA for the evaluation of breast lesions.

## 3 Conclusion

We demonstrated a new concept to treat solid tumors with PDT, characterized by the use of pulsed lasers to saturate the absorption of photosensitizers. This new therapeutic approach – named FLASH‐PDT – leads to a ratio of ROS in tumor/peritumoral tissues that is identical to the biodistribution ratio of the photosensitizer. This has profound implications in selectivity and, consequently, in the doses that can be used in PDT. Even when the photosensitizer tumor/peritumoral ratio is small, e.g., 4, it becomes possible to deliver a ROS concentration 2 times higher than the threshold dose for tissue necrosis in the tumor and 2 times lower than that threshold dose to the nearby healthy tissues, in all the regions where the absorption is saturated. This enables very selective ablation of the tumor. A most remarkable feature of FLASH‐PDT is that the number of pulses, and not the energy of each pulse, determines the concentration of ROS. However, the energy of each pulse must be sufficient to saturate the absorption of the photosensitizer. This highlights the importance of using photosensitizers with high molar absorption coefficients in the red/near‐infrared, such as phthalocyanines and bacteriochlorins. The saturation of the absorption of these families of photosensitizers for laser pulse fluences above 0.5 mJ cm^−2^ and their absorption in a spectral region with an optical penetration depth of ≈2 mm in human tissues, enables deep selective tumor necrosis in frontal illumination. For example, redaporfin with *C* ^0^_loc_ = 2.5 µm, *H* = 50 J cm^−2^, and *L*_0_ = 17 mJ cm^−2^, enables selective tumor necrosis to a depth of 7 mm. In view of the limits of radiant exposure for pulsed lasers, the weak red absorption of porphyrin derivatives cannot be saturated without significant thermal effects. If we are willing to accept some thermal effects at the surface of the tumor, we can also increase the depth of necrosis. For example, redaporfin with *C*_loc_ = 2 µm and *H* = 400 J cm^−2^ with *L*_0_ = 100 mJ cm^−2^, gives *d*_n_ = 10 mm. This laser pulse fluence should produce thermal effects on the surface of the tissue, but it is nevertheless much lower than that typically employed in tattoo removal: *L*_0_≈10 J cm^−2^.^[^ ^45^ ^]^ *Proper selection of the laser pulse fluence and of the number of pulses allows FLASH‐PDT to selectively ablate tumors and increase tumor necrosis depths*.

Good photosensitizers are not toxic in the dark. This suggests that they could be used at high doses without adverse effects. In reality, the doses are often limited by damage to healthy tissues and vital organs in the tumor margins as a result of their illumination, because of the modest selectivity of photosensitizers to cancer cells. FLASH‐PDT overcomes this limitation. We showed that the maximum light dose to treat ≈7 mm diameter subcutaneous CT26 tumors 15 min after i.v. administration of 0.75 mg kg redaporfin can be increased from the 50 J cm^−2^ of CW‐PDT to at least 432 J cm^−2^ in FLASH‐PDT, and make a statistically significant increase in survival. The dose of the photosensitizer can be similarly increased. We showed that redaporfin maximum tolerated dose can be increased from 0.75 to 15 mg kg^−1^, with a concomitant increase of the light dose from 50 to 160 J cm^−2^, to increase survival and obtain cures of mice with orthotopic 4T1 tumors. Additionally, this treatment of the primary tumor in the mammary gland was associated with the control of lung metastases, suggesting that this is an immune‐stimulating therapy. *FLASH‐PDT allows for order‐of‐magnitude increases of light or drug doses without adverse effects, with consequent increase in tumor responses*.

FLASH‐PDT enables the selective treatment of large tumors even when the photosensitizer's tumor‐to‐peritumoral tissue ratio is small. It overcomes two major limitations of PDT: selectivity and depth of treatment. PDT using pulsed lasers and photosensitizers with saturable absorptions within the phototherapeutic window is poised to drive a resurgence of PDT in oncology. The identification of pulse number as a determinant of lethal ROS levels, reported here for the first time, is expected to fundamentally influence the clinical development of FLASH therapies.

## 4 Experimental Section

### Theory of FLASH‐PDT

The derivation of the equations required to calculate [ROS]_c_ and [ROS]_f_ is presented in the Supporting Information.

### Liver Necrosis

The animal experiments were approved by the Portuguese Animal Health Authority (DGAV authorization 0421/000/000/2020), carried out in accordance with relevant guidelines and regulations, and in compliance with the ARRIVE guidelines. The BALB/c mice (Charles River Laboratories) were ≈10 weeks old (≈20 g) at the beginning of all experiments.

Under anesthesia, the skin just above the liver was depilated, and a spot with 1.3 cm in diameter was illuminated with a CW (Omicron) or a pulsed laser (Vevo LAZR‐X photoacoustic tomography equipment) at 750 nm. The CW laser was set to 105 mW cm^−2^, and the illumination lasted for 286 s. The 20 Hz pulsed laser illumination was performed in three conditions: 750 s for 2 mJ cm^−2^ pulses, 246 s for 6 mJ cm^−2^ pulses, or 91 s for 16.5 mJ cm^−2^ pulses. In all cases, the light dose delivered was 30 J cm^−2^. Three days after the photoactivation of redaporfin, the animals were anesthetized with isoflurane and then sacrificed by cervical dislocation. Four animals were used in each of the 3 groups.

### Tumor Models

CT26 cells (ATCC Cat# CRL‐2638) and 4T1 cells (ATCC Cat# CRL‐2539) were cultured in Dulbecco's Modified Eagle's medium supplemented with 10% heat‐inactivated fetal bovine serum, 100 U mL^−1^ penicillin, and 100 ng mL^−1^ streptomycin. CT26 was established by s.c. injection of 350 000 CT26 cells in the right flank of the mice. PDT was performed when the tumors largest diameter reached ≈5 mm, whereas in a second group of experiments tumor illumination was performed when tumors reached ≈7 mm. Briefly, redaporfin (0.75 mg kg^−1^) was administered via the tail vein, and 15 min later, tumors were illuminated using a pulsed laser at 750 nm (Vevo LAZR‐X) with a pulse fluence set at 24 mJ cm^−2^.

4T1 tumors were established by orthotopical injection of 20 000 cells in the fourth right abdominal mammary fat pad of the mice. PDT was performed when the tumors largest diameter reached 3.5–4.6 mm, typically 8–9 days after the inoculation. Redaporfin (15 mg kg^−1^) was administered via the tail vein, and tumor illumination was performed 15 min later using a pulsed laser at 750 nm (Vevo LAZR‐X), with a pulse fluence set at 11 mJ cm^−2^. Mice were covered with an opaque black fabric with a 1.13 cm^2^ hole over the tumor region, and a diffuser glass was placed over the 4T1 tumors to achieve more homogeneous light distribution.

### Magnetic Resonance Imaging

Acquisitions were performed on a 9.4T MR preclinical scanner (Bruker Biospec, Billerica MA) operated with ParaVision (v6.0.1). A volume resonator 1H transmit‐receive volume coils with an inner diameter 40 mm and 75 mm outer diameter. Morphological images of spleen and tumor were acquired with T2‐weighted TurboRARE in axial orientation, with the following parameters: TE/TR = 33.00/4200 ms, FOV = 30.0*30.0 mm, acquisition matrix = 200*200, averages = 6, resolution 0.150*0.150 mm, 40 continuous slices with 1.0 mm thick, with respiratory trigger. For high‐resolution lung images, a 1H transmit only resonator with an inner diameter 72 mm was used with 1H receive‐only mouse brain surface coil. The T2‐weighted turboRARE sequence adopted the following parameters: TE/TR = 23/976.799 ms, FOV = 30.0*30.0 mm, acquisition matrix = 320*320, averages = 8, echospacing = 7.667 ms, resolution 0.094*0.094 mm, 13 coronal continuous slices with 0.8 mm thick and respiratory trigger. The animals breathing rate and body temperature were monitored throughout the imaging procedures (SA Instruments). For quantitative MRI imaging processing, PMOD and View Tool (PMOD 4.205; PMOD Technologies; Zürich, Switzerland; RRID:SCR_016547) were used to delineate volumes of interest (VOIs).

### Statistical Analysis

The data were presented as means with SD, as noted in each case. All values of *n* were provided. One‐way Anova was used for comparisons between different laser pulse energies in the liver necrosis experiment, while two‐way Anova was used to compare mean tumor growth across different treatment groups. Survival analysis was performed using the Kaplan‐Meier estimator, and statistical significance between different treatment groups was assessed with long‐rank (Mantel‐Cox) test. *P* < 0.05 was considered to indicate a statistically significant difference; *P* < 0.05, *P* < 0.01, and *P* < 0.001 were indicated with single, double, and triple asterisks, respectively.

## Conflict of Interest

The authors declare no conflict of interest.

## Supporting information



Supporting Information

Supporting Information

## Data Availability

The data that support the findings of this study are available from the corresponding author upon reasonable request.
