# Supplementary material for: Flash Photodynamic Therapy – How the Saturation of Photosensitizer Absorption Enables Selective and Deeper Tumor Treatments
Source: Adv Sci (Weinh). 2025 Nov 16;13(1):e13199. doi: 10.1002/advs.202513199 (PMC12767129; doi:10.1002/advs.202513199)
Supplement: Supplementary file 2 — Supporting Information [file ADVS-13-e13199-s001.docx]

Supporting Information

Flash Photodynamic Therapy – How the saturation of photosensitizer absorption enables selective and deeper tumor treatments

Luis G. Arnaut*, Fábio A. Schaberle, José Sereno and Lígia C. Gomes-da-Silva

*Corresponding author: [**lgarnaut@ci.uc.pt**](mailto:lgarnaut@ci.uc.pt)

Excel files with FLASH-PDT calculations implemented for redaporfin, IR700 and BPD-MA are provided. An image of a file is presented at the end of the Supporting Information. The detailed derivation of the equations employed in the main text is presented below.

**1. Solution of the diffusion equation**

The oxygen diffusion equation with first-order oxygen consumption (*k*_1_) in Krogh’s model is^[1]^

$D\left( \frac{\partial^{2}C}{\partial r^{2}}+\frac{1}{r}\frac{\partial C}{\partial r} \right)-k_{1}C=0$ (1)

where *D* is the diffusion coefficient of O_2_ in the tissue (*D* = 2x10^–5^ cm^2^ s^–1^)^[2]^ and *C* is [O_2_]. Kirkpatric’s steady-state solution involves modified Bessel functions of the first and second kind of order zero, *I*_0_ and *K*_0_, and of the order one, *I*_1_ and *K*_1_, respectively, which are implemented in popular spreadsheets. The steady-state solution is^[1]^

$\left[ O_{2} \right]_{ss}=AI_{0}\left( R \right)+BK_{0}\left( R \right)$ (2a)

with

$A=\alpha C_{0}\frac{K_{1}\left( R_{1} \right)}{I_{1}\left( R_{1} \right)\left[ \frac{K_{1}\left( R_{1} \right)}{I_{1}\left( R_{1} \right)}I_{0}\left( R_{0} \right)+K_{0}\left( R_{0} \right) \right]}$ (2b)

$B=\alpha C_{0}\frac{1}{\frac{K_{1}\left( R_{1} \right)}{I_{1}\left( R_{1} \right)}I_{0}\left( R_{0} \right)+K_{0}\left( R_{0} \right)}$ (2c)

where *α* is the tissue/capillary substrate partition coefficient and the adimensional constants are related to the distances

$R=\sqrt{\frac{k_{1}}{D}}r R_{0}=\sqrt{\frac{k_{1}}{D}}r_{0} R_{1}=\sqrt{\frac{k_{1}}{D}}r_{1}$ (2d) The radii of the capillary and of the surrounding tissue are *r*_0_ and *r*_1_, respectively, and *r* is the distance at which [O_2_]_ss_ is calculated provided that *r*_0_ ≤ *r* ≤ *r*_1_. The radius of the surrounding tissue *r*_1_ is conveniently defined as half the intercapillary distance. This will naturally depend of the type of tissue. Intercapillary distance between 50 µm and 120 µm in tumors have been measured.^[3]^ The values used in Krogh’s model range from 2.5 to 10 µm for *r*_0_, and from 18 to 60 µm for *r*_1_.^[4]^ Here we will use *r*_0_ =3 µm and *r*_1_=60 µm.

Oxygen partial pressure (pO_2_) ranges from 14 mm Hg in a blood vessel to less than 5 mm Hg at 80 µm from the nearest blood vessel, which can be taken as the onset of hypoxia.^[5]^ pO_2_ values can be converted to [O_2_] using the conversion factor 10.1 µM/kPa (i.e., 1.34 µM/(mm Hg).^[6]^ Pogue and co-workers measured oxygen partial pressure values averaging 16 mm Hg (~20 µM) in highly oxygenated regions and 3.2 mm Hg (~4 µM) in hypoxic regions.^[7]^ Zhu and co-workers estimated [O_2_]≈40 µM in highly oxygenated tissues.^[4]^ ^[8]^ Here we will adopt [O_2_]_0_ =30 µM just outside the capillaries, which is considered the lower limit for physoxia.^[9]^ This adoption circumvents the need to explicit a value for *α*.

**2. Oxygen consumption rates**

The metabolic and photodynamic contributions to the rate constant of oxygen consumption in tissues can be written

$k_{1}^{'}=k_{PDT}^{'}+k_{met}^{'}$ (3)

The metabolic consumption rate of oxygen in known, *k’*_met_≈1.7 µM s^–1^,^[2]^ and can be expressed in terms of a first-order rate constant using a “typical” concentration of O_2_ in tissues, [O_2_]_0_=30 µM. This leads to the first-order rate constant for metabolic consumption of oxygen *k*_met_≈0.057 s^–1^.

The photodynamic rate of consumption of oxygen is the product of the rate of absorption of photons by the singlet oxygen quantum yield (_∆_) and by the fraction of singlet oxygen molecules that react with biological targets B^[10]^

$k_{PDT}^{'}=v_{h\nu}\Phi_{\Delta}\Phi_{B}$ (4a)

with

$v_{h\nu}=2300\varepsilon_{max}C_{loc}\frac{I_{loc}}{N_{A}E_{h\nu}}$ (4b)

where *C*_loc_ is the local concentration of the photosensitizer, the local irradiance *I*_loc_ is expressed in J cm^–2^ s^–1^_,_ and the rate of photon absorption *v*_h_ is expressed in M s^–1^. The definition of _∆_ is

$\Phi_{\Delta}=\Phi_{T}\frac{k_{q}\left[ O_{2} \right]}{k_{\mathrm{TS}}+k_{q}\left[ O_{2} \right]}\cong\Phi_{T}\tau_{T}k_{q}\left[ O_{2} \right]$ (5)

where the reciprocal of the triplet lifetime is 1/τ_T_ = (*k*_TS_ + *k*_q_[O_2_]). In tissues, τ_T_≈5 µs, *k*_q_≈10^9^ M^–1^ s^–1^ and [O_2_] is in the micromolar range. Under these conditions, we obtain reasonable estimates for _∆_ considering that τ_T_ is approximately constant. In fact, *τ*_T_ changes by less than 10% when* *_T_ ≈ 5 µs and [O_2_] varies within the range of concentrations typically found in tissues.

Literature estimates of the fraction of ^1^O_2_ species that reacts with biological targets B,

$\Phi_{B}=\frac{k_{B}\left[ B \right]}{k_{\Delta}+k_{B}\left[ B \right]}$ (6)

rather than decay back to ^3^O_2_, were based on a short singlet oxygen lifetime, _∆_ = 1/(*k*_∆_+*k*_B_[B]) = 100 ns in cells.^[2]^ Using this value, it was estimated that more than 95% of ^1^O_2_ in generated in cells reacted with biomolecules. However, single-cell experiments showed that _∆_≈3 µs inside cells.^[11]^ A better estimate of the reactivity of ^1^O_2_ in cells also needs to consider that the partition coefficient of O_2_ between phospholipid membranes and PBS is ~3 at 37 °C,^[12]^ and that ~70% of the content of a mammalian cell is water. This means that 56% of the oxygen molecules are in lipid domains and 44% are in aqueous domains of cells. This distinction is important because the lifetime of ^1^O_2_ in water is 3.5 µs (*k*_∆_^w^ = 2.87x10^5^ s^–1^) and in hydrocarbons is 20 µs (*k*_∆_^h^ = 5.0x10^4^ s^–1^).^[11b]^ Hence, the fractions of ^1^O_2_ in lipid and in aqueous domains that react with biomolecules are 0.85 and 0.14, respectively. Given the relative number of ^1^O_2_ species in these two domains, we estimate that the global fraction of ^1^O_2_ species that reacts with biomolecules is 0.54. Taking this fraction together with Equations (4) and (5), we obtain the first-order photodynamic rate of consumption of oxygen under CW illumination

$k_{PDT}^{c}={1240\varepsilon_{max}C_{loc}\frac{I_{loc}}{N_{A}E_{h\nu}}\Phi}_{T}\tau_{T}k_{q}$ (7)

Pogue and co-workers also showed that the highly oxygenated regions had an average drop of 4 mmHg after PDT with 24 J cm^–2^ at 200 mW cm^–2^, which could not be matched by the hypoxic regions. The drop to 4 mmHg, and not to lower pressures, is consistent with the photosensitizer triplet state lifetimes (*τ*_T_) in tissues in the 1 to 5 µs range and with an oxygen quenching rate constant *k*_q_≈10^9^ M^–1^ s^–1^.^[13]^ The fraction of triplet states quenched by oxygen calculated with *τ*_T_≈5 µs, *k*_q_≈10^9^ M^–1^ s^–1^ and [O_2_]≈4 µM is less than 2%. When [O_2_] approaches 4 µM, the contribution of PDT to deplete O_2_ molecules becomes negligeable and, consequently, this process no longer increases oxidative stress.

The same principles and expressions apply for pulsed-laser irradiation under non-saturation conditions, except that the irradiance that appears in the rate of photon absorption *k*^0^_PDT_, Equation (7), must be replaced by the average irradiance given by

$I_{p}=L_{p}p_{r}$ (8)

where *L*_p_ is the energy density per pulse (in units of J cm^–2^) and *p*_r_ is pulse repetition rate *p*_r_ = *n*_p_/∆*t* (in Hz).

The rate of photon absorption under pulsed-laser illumination in saturation conditions (FLASH-PDT) is limited by the concentration of the photosensitizer because the number of triplet states after each laser pulse is given by the product *C*_loc__T_. Hence, the first-order photodynamic rate of consumption of oxygen is

$k_{PDT}^{f}={0.54C}_{loc}\Phi_{T}\tau_{T}k_{q}p_{r}$ (9)

This can be calculated with an estimate of the local concentration of the photosensitizer in the illuminated tissue, which is usually in the µM range.

**3. Rates of ROS generation**

The rate of ROS generation must be identical to the rate of oxygen consumption by PDT. Under CW laser illumination and no photobleaching (*C*_loc_ = constant) this is, from Equation (7),

$\frac{\Delta\left[ \mathrm{ROS} \right]_{c}}{\Delta t}=k_{PDT}^{c}\left[ O_{2} \right]$ (10)

If *C*_loc_ remains constant during the illumination time ∆*t*, the total amount of ROS produced per unit volume of tissue in CW-PDT is^[14]^

$\left[ \mathrm{ROS} \right]_{c}= k_{PDT}^{c}\left[ O_{2} \right]\Delta t$ (11)

Similarly, for flash-PDT,

$\frac{\Delta\left[ O_{2} \right]}{\Delta t}=p_{r}C_{loc}\Phi_{T}{\tau_{T}k}_{q}\left[ O_{2} \right]=k_{PDT}^{f}\left[ O_{2} \right]$ (12)

and

$\left[ \mathrm{ROS} \right]_{f}= k_{PDT}^{f}\left[ O_{2} \right]\Delta t=C_{loc}\Phi_{T}{\tau_{T}k}_{q}\left[ O_{2} \right]n_{p}$(13)

where we made use of the definition of the number of pulses. This holds true until the attenuation of light fluence with the depth of penetration in tissues makes the local fluence drop below *L*_sat_. At depths greater than this, [ROS] is determined by the parameters in Equations (7) and (8).

**4. Attenuation of light in tissues**

Light in the VIS/NIR is appreciably absorbed and scattered by tissues. Accordingly, the light penetration depth depends on absorption (*µ*_a_) and reduced scattering (*µ*_s_’) coefficients^[15]^

$\delta=\frac{1}{\sqrt{3\mu_{a}\left( \mu_{a}+{\mu'}_{s} \right)}}$ (14)

The reduced scattering coefficient of tissues decreases monotonically as the wavelength increases. For consistency in the comparison between different photosensitizers, we will adopt the empirical equation for the scattering behavior versus wavelength proposed by Jacques for soft tissues

${\mu'}_{s}=a\left( \frac{\lambda}{500 (nm)} \right)^{-b}$ (15)

where *a*=18.9 cm^–1^ is the value of *µ*_s_’ at 500 nm for liver and *b*=1.286 is the corresponding scattering power.^[16]^

The value of *µ*_a_ for a given tissue depends on the sum of the contributions from all absorbing chromophores in that tissue, but for a realistic tissue Jacques showed that *µ*_a_ is approximately constant in the 630–800 nm region.^[16]^ Skin absorption in the 620–800 nm range is *µ*_a_ = 0.37±0.12 cm^–1^.^[15]^ Most *µ*_a_ values for soft tissues (excluding brain) in this wavelength range are in the 0.07–1.5 cm^–1^ range.^[17]^ A review reporting absorption at ~630 nm of a variety of tissues shows that *µ*_a_ is generally in the 0.5–5 cm^–1^ range (excluding brain tissue).^[18]^ A narrower range at this wavelength, 0.35–3.2 cm^–1^, was reported more recently.^[19]^ Detailed analysis of light attenuation in a specific tissue will require the specific value of *µ*_a_ for that tissue but for the interest of comparing CW and pulsed light in PDT, it is reasonable to employ a typical value for the absorption of tissues in the 630-800 nm range. We employ *µ*_a_=0.6 cm^–1^ and additionally include the correction for *µ*_a_ due to the absorption of light by the photosensitizer present in the tissue: *µ*_a_=0.6+2.3*ε*_max_*C*_loc_, in cm^–1^. The numerical value comes from the classical definitions of the transmission of light through a pathlength *l* used in Physics, *T*=e^–^*^µl^*, and in Chemistry, *T*=10*^–εlC^*. The light penetration depth calculated with the values of *µ*_s_’ and *µ*_a_ discussed above is *δ*=1.9 mm at 630 nm and *δ*=2.2 mm at 750 nm, in good agreement with the values proposed by Tuchin and co-workers for these wavelengths: 1.7 and 2.2 mm, respectively.^[15]^

The optical penetration depth allows for the calculation of the attenuation of light with depth *d*

$L_{d}=L_{0}e^{-d/\delta}$ (16)

where *L*_0_ is the radiant exposure at the surface of the tissue exposed to air. The actual *L*_loc_ relevant for the calculation of [ROS] must take in consideration the backscatter of light

$L_{loc}=b_{s}L_{d}$ (17)

Backscatter augments the irradiance near the surface of the tissue because ~50% of the photons scattered in the tissue towards the tissue/air interface are reflected back into the tissue by total internal reflection.^[20]^ In the phototherapeutic window, the total diffuse reflectance can be approximately related to the attenuation of the photons in the pathlength 8*δ* ^[21]^

$R_{d}\approx e^{-\mu_{a}8\delta}$ (18)

and be used to calculate the backscattering coefficient^[20, 22]^

$b_{s}=3+5.1R_{d}-2e^{-9.7R_{d}}$ (19)

The expressions above are empirical fits to results of Monte Carlo calculations, and are only strictly valid for light in the phototherapeutic window and for a ratio between the refractive indexes of tissue versus air of 1.38. These conditions apply for the cases addressed in this work. For example, using Equations (18-19) we calculate *b*_s_=4.3 at 630 nm and the value generally adopted for this wavelength in *b*_s_=4.4.^[14]^

**5. Bleaching of photosensitizers**

The photodecomposition quantum yield is defined as

$\Phi_{pd}=\frac{initial rate of disappearance of photosensitizers}{initial rate of absorption of photons}=\frac{v_{C}}{v_{h\nu}}$ (20)

Assuming that the photosensitizer photodecomposition is first-order with respect to the photosensitizer concentration, we have

${v_{C}=-\frac{dC_{loc}}{dt}=k_{pd}C_{loc}=v_{h\nu}\Phi}_{pd}$ (21)

Making use of Equation (4b) for the rate of absorption of photons, we can express the photodecomposition rate constant explicitly in terms of photosensitizer parameters

$k_{pd}=2300\frac{\varepsilon_{max}\lambda}{N_{A}hc}\Phi_{pd}I_{loc}$ (22)

and of the irradiance. The constant applies, and *k*_pd_ is expressed in units of s^–1^, when *ε*_max_ is in [M^–1^ cm^–1^], *I*_loc_ in [W cm^–2^] and the other quantities are in SI units. Given Equation (21), the first-order photodecomposition of the photosensitizer concentration under constant irradiance is

$C_{loc}=C_{loc}^{0}e^{-k_{pd}t}$ (23)

where *C*_loc_^0^ is the initial concentration of the photosensitizer in the tissue.

Examples of _pd_ representative of reasonably photostable photosensitizers are presented in Table 1. Using the value for redaporfin, _pd_=1×10^–5^, we obtain *k*_pd_=2.3x10^–3^ s^–1^ at an irradiance of 130 mW cm^–2^, and a decrease by a factor of 0.4 of the initial redaporfin concentration after the delivery of 50 J cm^–2^. This means that photobleaching during PDT cannot be neglected.

The modification of Equation (11) to allow for the calculation of the total amount of [ROS] with photosensitizer bleaching under CW illumination requires the calculation of the integral

$\left[ \mathrm{ROS} \right]_{c}=1240\frac{\lambda\varepsilon}{hcN_{A}}\Phi_{T}{\tau_{T}k}_{q}\left[ O_{2} \right]I_{loc}C_{loc}^{0}\int_{0}^{t} e^{-k_{pd}t}dt$ (24)

assuming that [O_2_] is independent of time. We opted for a steady-state solution for oxygen concentration in tissues which is consistent with this assumption. This integral gives

$\left[ \mathrm{ROS} \right]_{c}=1240\frac{\lambda\varepsilon}{hcN_{A}}\Phi_{T}{\tau_{T}k}_{q}{\left[ O_{2} \right]_{ss}I}_{loc}C_{loc}^{0}\frac{\left( 1-e^{-k_{pd}t} \right)}{k_{pd}}$ (25)

Of note, given the limit

$\lim_{x\to0} \left( \frac{e^{ax}-1}{x} \right)=a$ (26)

when *k*_pd_0, Equation (25) tends to Equation (11), as it should. The value of [ROS]_c_ at *r*_1_=60 µm is calculated with and oxygen concentration that is twice the value calculated with Equation (2) at this distance, to account for oxygen diffusion from the equidistant capillary. When this value is larger than 30 µM, we make [O_2_]_ss_=30 µM.

In flash-PDT, the amount of photosensitizer remaining after each laser pulse is *C*_loc_ = *C*_loc_^0^ (1–_pd_). Hence, the fraction of photosensitizer remaining after *n*_p_ laser pulses is

$C_{loc}={C_{loc}^{0}\left( {1-\Phi}_{pd} \right)}^{n_{p}}$ (27)

For relatively stable photosensitizers, i.e., _pd_ ≤10^–4^ in solution, values of *n*_p_ = 4000, 8000 and 16000 give fractions of remaining photosensitizer of 0.67, 0.45 and 0.20, respectively. In these slowly varying conditions, it is reasonable to use *C*_loc_ at *n*_p_/2 and calculate the concentration of ROS after all the laser pulses as

$\left[ \mathrm{ROS} \right]_{f}=\Phi_{T}{\tau_{T}k}_{q}C_{loc}^{0}\left( 1-\Phi_{pd} \right)^{{n_{p}}/2}\left[ O_{2} \right]_{ss}n_{p}$ (28)

6. Image of the excel file with implementation of all the equations required to calculate [ROS]


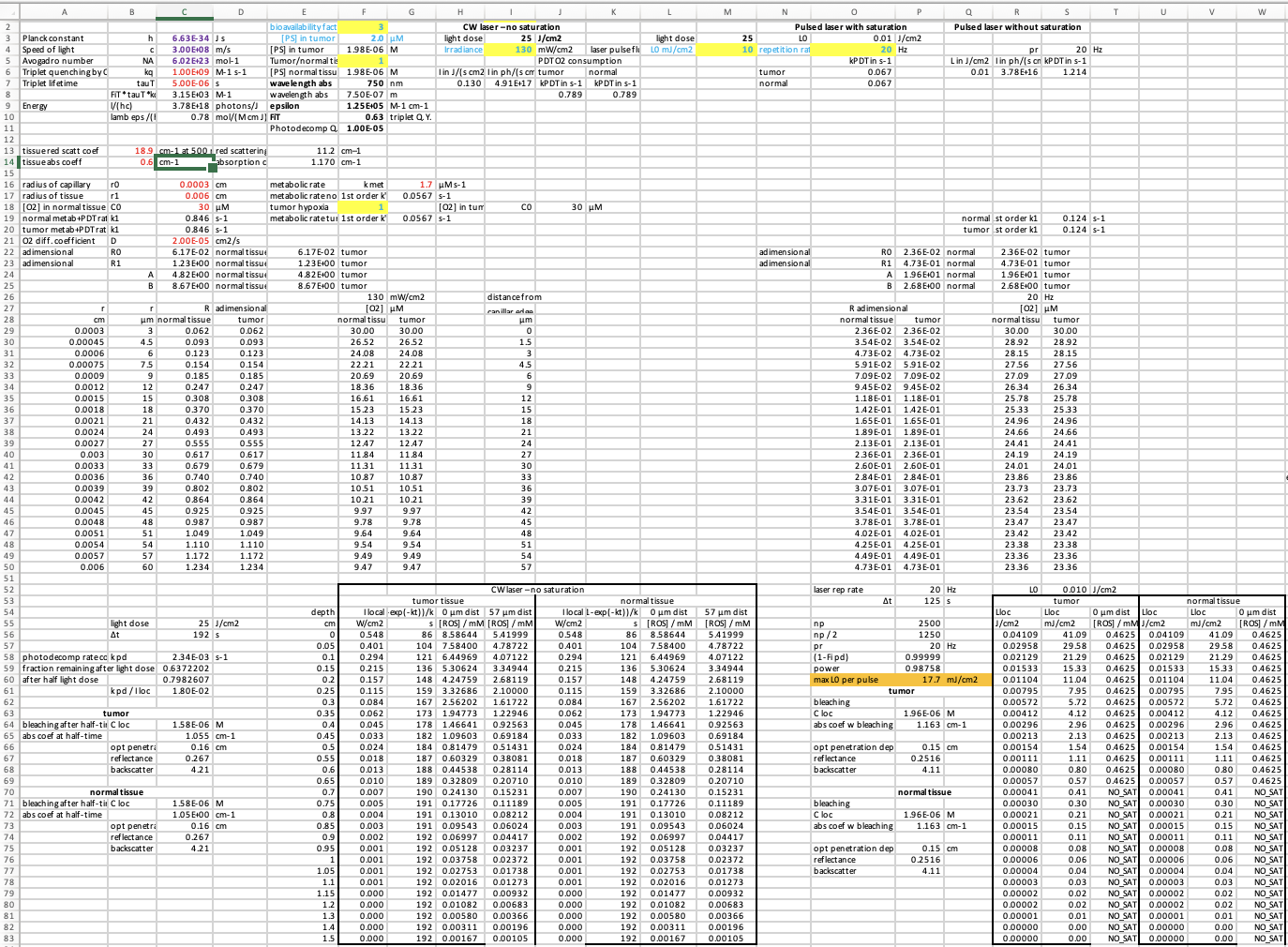


**7. Instructions to use the excel files to calculate [ROS] for various photosensitizers**

Three files are provided, formatted with the data for redaporfin, BDP-MA and IR700. They can be adapted for calculations with other photosensitizers with appropriate changes of the following parameters:

Molecular weight (g/mol) C1

Photosensitizer dose (mg/lg) F1

Bioavailabitity factor F2

Tumor/peritumoral tissue ratio F5

Wavelength of absorption (nm) F7

Molar absorption coefficient at the wavelength of absorption (M^–1^ cm^–1^) F9

Triplet quantum yield F10

Photodecomposition quantum yield F11

Pulse radiant exposure that saturates absorption (mJ/cm^2^) Q1

If the triplet lifetime is shorter than 5 µs, this will require an additional change:

Triplet lifetime (µs) C7

Rather than using the molecular weight, photosensitizer dose and bioavailability factor to calculate the concentration of the photosensitizer in the tumor, this value can be introduced directly

Concentration of photosensitizer in the tumor (µM) F2

Two important biological properties can be changed, if detailed information is available:

[O_2_] in normal tissue (µM) C18

Tumor hypoxia F18

(a higher value of tumor hypoxia lowers [O_2_] in tumors relative to normal tissues)

Finally, the data related to the illumination conditions must be adapted for each case:

Radiant exposure, or light dose (J/cm^2^) I1

Irradiance when a CW laser is used (mW/cm^2^) I4

Laser fluence when a pulsed laser is used (mJ/cm^2^) M4

Laser pulse repetition rate (Hz) O4

Seven sets of results are obtained with these data.

The first set of results refers to [O_2_] in normal (F29:F50) and in tumor (G29:G50) tissue at the corresponding distances from the closest capillary edge (I29:I50) under the conditions established for CW illumination.

The second set of results refers to [O_2_] in normal (R29:R50) and in tumor (R29:R50) tissue at the corresponding distances from the closest capillary edge (I29:I50) under the conditions established for pulsed illumination.

These data inform on whether oxygen depletion can be ignored or must be taken into consideration. Oxygen depletion must be considered if the calculated [O_2_] drops below half of its value at the capillary edge. This does not occur with pulsed light and repetition rates below 100 Hz, and oxygen depletion is not further considered in FLASH-PDT.

The third set of values refers to [ROS]_c_, more precisely to [ROS] under CW illumination in tumor (H56:H83 and I56:I83) at the corresponding depths from the surface (E56:E83). Two sets of results are presented for headlines “0 µm dis” and “57 µm dist”, which refer to calculations using [O_2_]_ss_ at the edge from the closest capillary or 57 µm from this edge. In the latter case, the concentration of [O_2_]_ss_ in G50 was multiplied by a factor of 2, to account for the presence of another capillary 120 µM from the center of the one considered in the calculations. *The values of [ROS]_c_ that must be considered to determine the depth of necrosis ([ROS]>0.7 mM) are those of the column (H56:H83 or I56:I83) with lower values, because they ensure that all cancer cells located up to that depth where exposed to [ROS]>0.7 mM*.

The fourth set of values refers to [ROS]_c_, more precisely to [ROS] under CW illumination in peritumoral (muscle) tissues (L56:L83 and M56:M83) at the corresponding depths from the surface (E56:E83). The interpretation is the same as above.

The fifth set of values refers to the local laser fluence (S56:S83) and [ROS]_f_ (T56:T83) under pulsed illumination in tumor tissues at the corresponding depths from the surface (X56:X83). The value of [ROS]_f_ is constant provided that the absorption is saturated. When the depth increases and the local laser fluence (S56:S83) drops below the (Q1) a warning message appears. For such depths, the value of [ROS]f must be taken from (Z56:Z83).

The sixth set of values refers to the local laser fluence (V56:V83) and [ROS]_f_ (W56:W83) under pulsed illumination in peritumoral (muscle) tissues at the corresponding depths from the surface (X56:X83). The interpretation is the same as above.

The seventh refers to [ROS]_f_ for pulsed laser when the photosensitizer absorption is no longer saturated, and presents data for tumor (Z56:Z83) and peritumoral (muscle) tissues (AB56:AB83) as a function of depth from the tissue surface (X56:X83). Only the values calculated for conditions of non-saturated absorption, which can be identified in columns T56:83 and W56:83, are physically meaningful.

[1] J. P. Kirkpatrick and M. W. Dewhirst, *Radiat. Res.* **2008**, *169*, 350-354.

[2] T. H. Foster, R. S. Murant, R. G. Bryant, R. S. Knox, S. L. Gibson and R. Hilf, *Radiat. Res.* **1991**, *126*, 296-303.

[3] a) Y. Yoshii and K. Sugiyama, *Cancer Res.* **1988**, *48*, 2938-2941; b) J. R. Less, T. C. Skalak, E. M. Sevick and R. K. Jain, *Cancer Res.* **1991**, *51*, 265-273.

[4] T. Zhu, C., B. Liu and R. Penjweini, *J. Biomed. Opt.* **2015**, *20*, 038001.

[5] G. Helmlinger, F. Yuan, M. Dellian and R. K. Jain, *Nat. Med.* **1997**, *3*, 177-182.

[6] R. Springett and H. M. Swartz, *Antioxid. Redox Signal* **2007**, *9*, 1295-1301.

[7] B. W. Pogue, R. D. Braun, J. L. Lanzen, C. Erickson and M. W. Dewhirst, *Photochem. Photobiol.* **2001**, *74*, 700-706.

[8] T. Sheng, Y. H. Ong, T. M. Busch and T. Zhu, C., *Proc. SPIE Int. Soc. Opt. Eng.* **2019**, *10860*, 108600V.

[9] S. R. McKeown, *Br. J. Radiol.* **2014**, *87*, 20130676.

[10] a) M. G. Nichols and T. H. Foster, *Phys. Med. Biol.* **1994**, *39*, 2161-2181; b) B. W. Pogue and T. Hasan, *Radiat. Res.* **1997**, *147*, 551-559.

[11] a) M. Westberg, M. Bregnhøj, A. Blásquez-Castro, T. Breitenbach, M. Etzerodt and P. R. Ogilby, *J. Photochem. Photobiol. A: Chem.* **2016**, *321*, 297-308; b) E. F. F. Silva, B. W. Pedersen, T. Breitenbach, R. Toftegaard, M. K. Kuimova, L. G. Arnaut and P. R. Ogilby, *J. Phys. Chem. B* **2012**, *116*, 445-461.

[12] M. N. Möller, Q. Li, M. Chinnaraj, H. C. Cheung, J. R. Lancaster Jr and A. Denicola, *Biochim. Biophys. Acta* **2016**, *1858*, 29232930.

[13] L. G. Arnaut and S. J. Formosinho, *Pure Appl. Chem.* **2013**, *85*, 1389-1403.

[14] Task Group Report of the General Medical Physics Committe, in *Photodynamic Therapy Dosimetry*, *Vol.88*  American Association of Physicists in Medicine, Medical Physics Publishing, **2005**.

[15] A. N. Bashkatov, E. A. Genina, V. I. Kochubey and V. V. Tuchin, *J. Phys. D: Appl. Phys.* **2005**, *38*, 2543-2555.

[16] S. L. Jacques, *Phys. Med. Biol.* **2013**, *58*, R37-R61.

[17] J. L. Sandell and T. Zhu, C., *J. Biophotonics* **2011**, *4*, 773-787.

[18] W.-F. Cheong, S. A. Prahl and A. J. Welch, *IEEE J. Quantum Electron.* **1990**, *26*, 2166-2185.

[19] J. Mobley, T. Vo-Dinh and V. V. Tuchin in *Optical properties of tissue*, *Vol. 1* (Ed. T. Vo-Dinh), CRC Press, **2014**, pp. pp.23-122.

[20] S. L. Jacques, *Photochem. Photobiol.* **1998**, *67*, 23-32.

[21] S. L. Jacques, *J. Biomed. Opt.* **2010**, *15*, 051608.

[22] S. L. Jacques, *Proc. SPIE* **2002**, *4612*, 59-68.
